# Preparation and Evaluation of the Fully Humanized Monoclonal Antibody GD-mAb Against Respiratory Syncytial Virus

**DOI:** 10.3389/fcimb.2019.00275

**Published:** 2019-07-31

**Authors:** Puyuan Tian, Yuqing Wang, Hui Liu, Yulu Yang, Xiaoli Wu, Hua Wei, Tingtao Chen

**Affiliations:** ^1^National Engineering Research Center for Bioengineering Drugs and the Technologies, Institute of Translational Medicine, Nanchang University, Nanchang, China; ^2^JiangXi University of Traditional Chinese Medicine, College of Basic Medicine, Nanchang, China; ^3^State Key Laboratory of Food Science and Technology, Nanchang, China

**Keywords:** respiratory syncytial virus (RSV), phage display technology, monoclonal antibody, inflammation, signal pathway

## Abstract

Respiratory syncytial virus (RSV) is the major cause of pulmonary and bronchial inflammation in infants, young children, and immunocompromised adults, but therapeutic options to control RSV are limited. In the present study a single chain antibody against RSV (GD-scFv) was screened using phage display library panning technology and a full-length monoclonal antibody (GD-mAb) was developed from GD-scFv based on the sequence encoding Ig V_H_ and Ig V_L_. The anti-RSV potential of GD-mAb was evaluated *in vitro* and in mice. Our results indicated that both GD-scFv (4.25 ± 2 nM) and GD-mAb (3.13 ± 0.89 nM) showed high binding capability and strong binding specificity to GD protein. GD-mAb effectively neutralized RSV and reduced the plaque number in a concentration-dependent manner through a plaque reduction neutralization assay. In mice, GD-mAb lowered the lung index and reduced the lung virus titer in the mouse lung (*p* < 0.05). Antibody treatment reduced the phosphorylated protein level in pathways of TLR4/NF-κB, MAPKs, and PI3K/Akt (*p* < 0.05) and correlated with an absence of pro-inflammatory factors TNF-α, IL-1β, and IL-6 in the mouse lung and serum (*p* < 0.05). In summary, these data suggest that GD-mAb may be an effective therapeutic agent for the treatment of RSV infections.

**Importance**

Currently, only a few therapeutic options are available to control respiratory RSV in humans. In this study, our group developed a full-length monoclonal antibody (GD-mAb) and reported a high binding specificity of the RSV surface glycoproteins G. Moreover, GD-mAb effectively neutralized RSV *in vitro*, and significantly lowered the lung index and reduced the lung virus titer in an infected mouse lung, which suggests that GD-mAb may serve as an effective antiviral agent for RSV infection.

## Introduction

A member of the *Paramyxoviridae*, respiratory syncytial virus (RSV) is regarded as the leading cause of viral lower respiratory tract infection in infants and young children worldwide (Weinberg, 2017). Studies indicate that RSV causes 33,100,000 episodes, 3,200,000 hospital admissions, and 59,600 in-hospital deaths each year in children younger than 5 years of age (Shi et al., 2017; Weinberg, 2017). To date, vaccination against RSV has been unsuccessful in young children, because of their immature immune system (Tripp, 2004; Fauci et al., 2008; Haynes et al., 2009). However for infants with a high risk of serious disease, the neutralizing antibodies motavizumab and palivizumab may be used (DeVincenzo et al., 2014; Murray et al., 2014). It is important to note that these antibodies therapeutics are not effective for the treatment of an active infection (Fauci et al., 2008) and for immune compromised patients (Varga and Braciale, 2002).

In previous studies, three glycoproteins (F, G, and SH) have been proven to play important roles in triggering a host protective immune response (Fauci et al., 2008; McLellan, 2015; Tian et al., 2018). The G protein is recognized as an important neutralizing antigen in inducing and modulating the host immune response to RSV infection (Murawski et al., 2010; Tian et al., 2018). Our group has evaluated the effect of truncated G protein (GD) delivered by live attenuated *Salmonella* as a vaccine against RSV, which represents a promising approach to protect the host against RSV (Tian et al., 2018). In the present study our group isolated a single chain antibody (GD-scFv) from a phage display library by panning the GD protein. We engineered the variable region of its heavy chain and the variable region of the light chain into the corresponding vectors (IgG and Igκ) to develop the full-length monoclonal antibody (GD-mAb) and evaluated its protective efficacy against RSV both *in vitro* and in mice.

## Methods

### Construction and Preliminary Screening of Single-Chain Antibody Fragment (scFv) Phage Antibody Library

The products of the scFv antibody gene library were inoculated into SB-A+ liquid medium (containing 100 mg/L ampicillin), incubated with shaking to OD_600_, reaching 0.9–1 at 37°C, and helper phage M13K07 (4 × 10^10^ pfu/100 mL) and kanamycin (70 mg/L) were added. After overnight culturing in SB-A+K+ liquid medium (containing 100 mg/L ampicillin and 70 mg/L kanamycin), it was centrifuged (8,000 g for 15 min, at 4°C) and the phage antibodies in the supernatant were precipitated with 80 g/L PEG 8000 and 60 g/L NaCl, and resuspended in PBS (containing 1% BSA and 10% glycerin). After centrifugation, the supernatants were obtained and the scFv phage antibody library was constructed and plated. The purified GD protein was coated in a 96-well plate overnight at 4°C. The next day, plates were washed with PBS, blocked with 5% BSA-PBS and incubated for 1 h at 37°C. The phagemid populations were added to the plate, and after incubation for 2 h at 37°C the plate was emptied and washed with 0.5% PBST (containing 0.5% Tween20) five times. Log phase *Escherichia coli* XL1-Blue (*E. coli TG1*) and M13K07 were then added. The phages were re-infected and were rescued after a 2 h incubation at 37°C. The panning process was repeated for four times.

### Isolation and Verification of the Affinity and Specificity of GD-scFv

In collaboration with Beijing Gegen Biotechnology Co., Ltd. (Beijing, China), we obtained the variable regions of the light chain (V_L_) genes and variable regions of the heavy chain (V_H_) genes, from the phage antibody library, using GD protein as antigen (Tian et al., 2018). The genes of V_H_ and V_L_ were engineered into vector pET-28a with a linker, and the protein GD-scFv was expressed using *E. coli BL21*. Ni–NTA affinity chromatography was applied here to purify GD-scFv (with a His tag) from periplasmic lysates, and its purity and molecular weight were tested using SDS-PAGE method.

The GD protein was then coated in the bottom of 96-well ELISA plates overnight at 4°C, and tenfold serially-diluted antibodies were prepared in 10% FBS and were added into the antigen-coated plates for 1 h at 37°C. Plates were washed three to five times with TBST and 100 μl 1:1,000 diluted anti-His antibody (Cell Signaling Technology, 2365P) was then added and was incubated for 1 h at 37°C. Plates were washed three to five times with TBST, and the second antibody with HRP was used. TMB substrate was used as the substrate for color development and plates were read at 450 nm.

To test the degree of specificity of GD-scFv, an irrelevant IgG (Bethyl Laboratories, A80-104P) was used as the antigen, and coated in the bottom of 96-well ELISA plates to test the non-specific affinity of GD-scFv. Kd was calculated by the equation Kd = 2[Ab′]_t_-[Ab]_t_, where [Ab′]_t_ refers to the scFv concentration at OD_450_ for the half concentration of GD protein coated wells, while [Ab′]_t_ refers to the scFv concentration at OD_450_ for the full-strength concentration of GD protein coated wells.

### Construction and Verification of the Affinity and Specificity of GD-mAb

Genes encoding Ig V_H_ and Ig V_L_ from GD-scFv were cloned into IgG heavy- and light-chain expression vectors (IgG and Igκ), the GD-mAb was expressed by transfection of 293T cells and was purified as described previously (Tiller et al., 2008; Huang et al., 2013). The GD protein and irrelevant IgG (Bethyl Laboratories, A80-104P) were then coated in the bottom of 96-well ELISA plates to test the affinity and degree of specificity of GD-mAb.

### Evaluation of the Neutralization Effect of GD-mAb on RSV *in vitro*

Vero cells (African green monkey kidney fibroblasts, Chinese Academy of Sciences Cell Bank, GNO10) were serially passaged in Dulbecco's Modified Eagle Medium (DMEM) (Gibco BRL, Grand Island, NJ, USA) supplemented with 5% fetal bovine serum (FBS, Gibco BRL, Grand Island, NJ, USA), 100 units of penicillin G, 100 mg/mL of streptomycin, 0.2% sodium bicarbonate, and 2 mM L-glutamine. The RSV long strains (kindly donated by Dr. Xiaoming Liang, Boya Biology Co., Ltd.) were passaged more than three times in Vero cells in our laboratory and the virus stocks were stored at −80°C.

GD-mAb was diluted in DMEM containing 10% FBS at different concentrations with 50 PFU of RSV and incubated for 1 h at 37°C. The mixture was then added into 12-well plates at 2 × 10^4^ cells/well and incubated at 37°C for 45 min. The cells were then washed and DMEM containing 1% methylcellulose was added, the cells were incubated at 37°C for 4 days and fixed with 10% formaldehyde for 30 min. Finally, hematoxylin and eosin were used to count the white plaques on the wash plate.

### Animal Study

Eight-week-old female BALB/c mice, provided by Hunan Silaike Jingda Laboratory Animal Co., Ltd. (Changsha, Hunan, China), were kept in the animal facility under standard conditions (humidity 50 ± 15%, temperature 22 ± 2°C, 12/12 light-dark cycle) and were fed a standard diet. The animals were randomized into eight groups (*n* = 14 per group). Three mice were sacrificed on day 3 for viral titer tests only, eight mice were sacrificed on day 7 for all the tests; three mice were sacrificed on day 11 for viral titer tests only. The eight groups were: (1) the control group (C); (2) the group challenged with RSV (M); (3) the group pre-treated with purified GD-mAb (3 mg/kg) and then challenged with RSV (PH); (4) the group pre-treated with purified GD-mAb (1.5 mg/kg) and then challenged with RSV (PL); (5) the group challenged with RSV and then treated with ribavirin (0.05 g/kg per day, H20056707, Sichuan Baili Pharmaceutical Co., Ltd.) (R); (6) the group challenged with RSV and then treated with purified GD-mAb (0.032 mg/kg) (TL); (7) the group challenged with RSV and then treated with purified GD-mAb (0.16 mg/kg) (TM); (8) the group challenged with RSV and then treated with purified GD-mAb (0.32 mg/kg) (TH). Mice received 10^7^ PFU/mL RSV (100 μl) after fluorothane anesthesia for 3 days, and purified GD-mAb was injected intravenously at a specific time. After inoculation, the mice were killed, and the lungs were removed for further analysis.

The present study was approved by the Ethical Committee of the Second Affiliated Hospital of Nanchang University (Nanchang, China) and all methods were performed in accordance with the approved guidelines.

### Lung Index

After the final exposure to the RSV infection described in section Animal Study, mice were weighed sacrificed, and the whole lung was taken from the chest. Lung tissue was rinsed twice with pre-cooled physiological saline and then surface water was dried and the lungs were weighed. Lung index = [lung weight (g)/body weight (g)] × 100%.

### Viral Titers

Viral titers in the lungs of RSV-infected mice were determined as described elsewhere (Haynes et al., 2002). Briefly, lungs were aseptically removed from the mice on days 3, 7, and 11 after infection and were stored at −80°C until assayed. Lungs were weighed and individual lung samples were homogenized in 1 ml of cold, sterile PBS, and tenfold serial dilutions (in serum-free DMEM) of the lung homogenates were added to confluent Vero cell monolayers in 12-well plates. After adsorption for 1 h at 37°C, cell monolayers were overlaid with tissue culture DMEM containing 1% methylcellulose (Sigma, Chem- ical Co.), incubated at 37°C for 4 or 5 days, and viral plaque formation was enumerated using hematoxylin and eosin staining.

### Histopathological Analysis

The excised samples of the lungs were fixed in 4% paraformaldehyde, embedded in paraffin and processed for histopathological analysis. All samples were cut into 5 μm sections, rehydrated with xylene and declining grades of ethanol for 5 min, and washed three times using PBS for another 5 min, the lung slides were used for hematoxylin and eosin (H&E) staining. A histopathological evaluation was performed to evaluate the integrity of the alveolar structure, the infiltration of the alveolar cavity and interstitial inflammatory cells, by a histopathologist with no prior knowledge of the identity of the samples.

### Western Blot Analysis

Lung tissues were homogenized and extracted using normal sodium, and then centrifuged at 8,000 g for 25 min. Protein concentrations were measured and proteins were resolved using SDS (sodium dodecyl sulfate) polyacrylamide gel electrophoresis (Hu et al., 2016; Zeng et al., 2017). Then proteins were electro transferred to polyvinylidene difluoride membranes and blocked using 5% bovine serum (BSA) in TBST for 1.5 h at RT. Membranes and primary antibodies were then co-incubated overnight at 4°C, washed with TBST buffer, and then appropriate amounts of secondary antibody conjugated with HRP were added for another 1 h, at RT. Primary antibodies anti-mouse β-actin (1:1,000; Cell Signaling Technology, 4970S), anti-mouse NF-kB (1:1,000; Cell Signaling Technology, 8242S), anti-mouse TLR4 (1:1,000; Santa Cruz Biotechnology, sc-293072), anti-mouse p-p38 (1:1,000, CapitalBio Corporation, bs-5477R), anti-mouse p-ERK (1:2,000, Cell Signaling Technology, 4370), anti-mouse PI3K (1:1,000, CapitalBio Corporation, bs-5570R), and anti-mouse p-AKT (1:2,000, Cell Signaling Technology, 4060) were used in the present study.

### RNA Preparation and Quantitative PCR

For the evaluation of cytokine mRNA expression levels, total RNA from the lung was prepared by adding TRIzol reagent (Gibco BRL, Grand Island, NJ, USA) according to the manufacturer's protocol. Total RNA of the samples was extracted, and the purity was tested using a NanoDrop 2000 spectrophotometer (Thermo Fisher Scientific) and 1 μg of total RNA from each group was reverse transcribed to cDNA using a commercially available kit (Takara). Quantitative real-time PCR was performed with the 7900HT fast real-time PCR system (ABI) using 2 × SYBR Green master mix (Takara). The relative expression levels of the target genes were analyzed using the 2^−ΔΔ*Ct*^ method for polymerase activation with the following primers (Q-PCR IL-1β: sense primer 5′-GTGTCTTTCCCGTGGACCTTC-3′, antisense primer 5′ -TCATCTCGGAGCCTGTAGTGC-3′; Q-PCR TNF-α: sense primer 5′ -GTGGAACTGGCAGAAGAGGCA-3′, antisense primer 5′ -AGAGGGAGGCCATTTGGGAAC-3′; Q-PCR IL-6: sense primer 5′- GAAATCGTGGAAATGAG-3′, antisense primer 5′-GCTTAGGCATAACGCACT-3′; Q-PCR GAPDH: sense primer 5′ -CTCGTGGAGTCTACTGGTGT-3′, antisense primer 5′ -GTCATCATACTTGGCAGGTT-3′).

### Quantitation of TNF-α, IL-1β, and IL-6

TNF-α (Cloud Clone Corp, SEA133Mu), IL-1β (Cloud clone crop, SEA563Mu), and IL-6 (Cloud Clone Corp, SEA079Mu) levels in serum were analyzed using a capture enzyme-linked immunoassay kit, in accordance with the manufacturer's instructions (Cloud Clone Corp).

### Data Analysis

Statistical analysis was performed using Prism software version 7.0 (GraphPad Software, San Diego, CA, USA). Data are shown as means ± SD. Statistical significance was analyzed using one-way analysis of variance (ANOVA) followed by Tukey's multiple comparison tests. Error probabilities of *p* < 0.05 were considered statistically significant.

## Results

### Characteristics of GD-scFv Isolated From Phage Display Libraries

**Figure 1:**
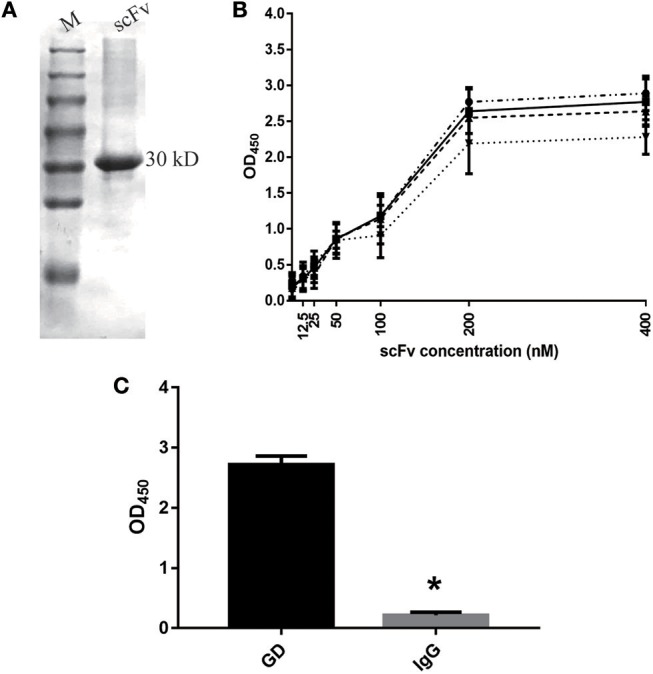
Characteristics of GD-scFv isolated from phage display libraries. **(A)** The purity and molecular weight of GD-scFv using SDS-PAGE (30 kDa). **(B)** Measurement of the Kd between GD- scFv using ELISA. The calculated Kd for scFv-GD was 6.25 nM using antigen concentrations of 2 μg/ml (-··) and 1 μg/ml (—), 4.72 nM using antigen concentrations of 1 and 0.5 μg/ml (- - -), or 2.15 nM using antigen concentration of 0.5 and 0.25 μg/ml (…). **(C)** Evaluation of the specificity of scFv-GD using ELISA method. Results are presented as the mean ± SD. ^*^P < 0.05 vs. control group.

As shown in Figure 1A, our results indicate that we successfully purified the GD-scFv protein (about 30 Kd). We then used Beatty's equation for Kaff [1/2(2[Ab′]_t_-[Ab]_t_), where [Ab′]_t_ refers to GD-scFv concentration at half the maximal OD (OD_50_) for GD-coated wells at half concentration, and [Ab]_t_ refers to GD-scFv concentration at OD_450_ for GD-coated wells at 100% concentration] to calculate the affinity of GD-scFv, and found that the Kd of GD-scFv was 4.25 ± 2 nM (Figure 1B). Moreover, the low binding reactivity of a non-specific IgG with GD-scFv suggested that GD-scFv possessed a high specificity on GD protein (Figure 1C).

### Evaluation of Properties of GD-mAb *in vitro*

**Figure 2:**
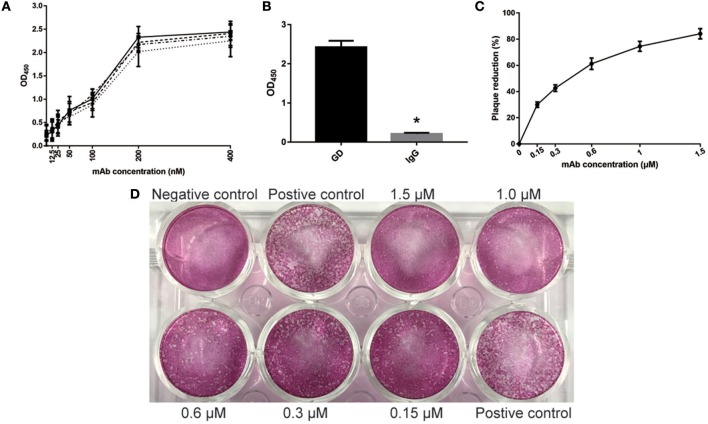
Evaluation of properties of GD- mAb *in vitro*. **(A)** Measurement of GD- mAb using ELISA. The calculated Kd for mAb-GD was 4.01 nM using antigen concentrations of 2 μg/ml (-··) and 1 μg/ml (—), 3.41 nM using antigen concentrations of 1 and 0.5 μg/ml (- - -), or 2.23 nM using antigen concentration of 0.5 and 0.25 μg/ml (…). **(B)** Evaluation of the specificity of GD- mAb using the ELISA method. **(C,D)** Evaluation of the neutralization effect of GD- mAb on respiratory virus using Vero cells. Results are presented as the mean ± SD. ^*^P < 0.05 vs. control group.

Similar to GD-scFv, a high affinity (3.13 ± 0.89 nM) and a high specificity of GD-mAb for the GD protein was observed (Figures 2A,B). The plaque reduction neutralization test was used to test the sensitivity and the neutralizing effect on the virus. Our results indicate that the addition of GD-mAb reduces the plaque number in a concentration-dependent manner (Figures 2C,D).

### Effects of GD-mAb on Histopathology and Viral Replication in Lung Tissue

**Figure 3:**
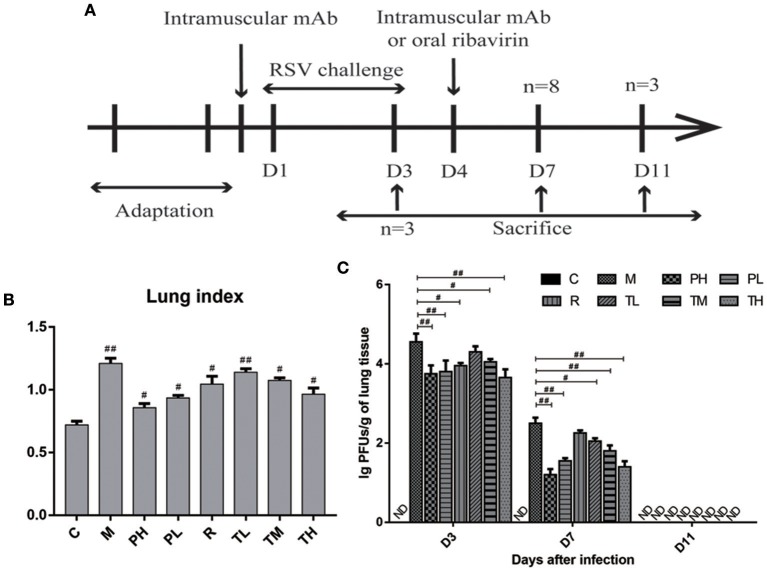
Effects of GD- mAb on histopathology and viral replication in lung. **(A)** Experimental protocol for the evaluation of the effect of GD- mAb on the RSV-infection mouse model. **(B)** Lung index (D7, *n* = 3 in each group) (LI, lung wet weight/body weight) of each treatment group. **(C)** Comparison of lung viral titers in different groups (D3, *n* = 3 in each group, D7, *n* = 3 in each group, D11, *n* = 3 in each group). Results are presented as the mean ± SD. ^#^*P* < 0.05 vs. model group; ^##^*P* < 0.01 vs. model group.

To further evaluate the anti-RSV effect of GD-mAb, a mouse model was employed (Figure 3A). As shown in Figure 3B, both the treatment group (TL, TM, and TH) and the prevention group (PH and PL) significantly lowered lung index (lung wet weight/body weight) at 72 h after RSV infection. The PH group (0.85) showed the greatest effect, compared with the M group (1.21) (*p* < 0.01). In addition, we measured the lung virus titer in each group on days 3, 7, and 11, and found that both the treatment group (TL vs.TM vs. TH = 4.3 vs. 4.1 vs. 3.7) and the prevention group (PH vs. PL = 3.8 vs. 3.9) showed a sound anti-RSV effect on day 3 compared with the M group (4.6). On day 7, GD-mAb in the treatment group and the prevention group had significantly decreased virus numbers, especially in groups PH and TH (1.2 vs. 1.5) compared with the M group (2.5) (*p* < 0.01). On day 11 no virus was detected in any group (Figure 3C).

### Effects of GD-mAb on the Inflammatory Status of the Lung

**Figure 4:**
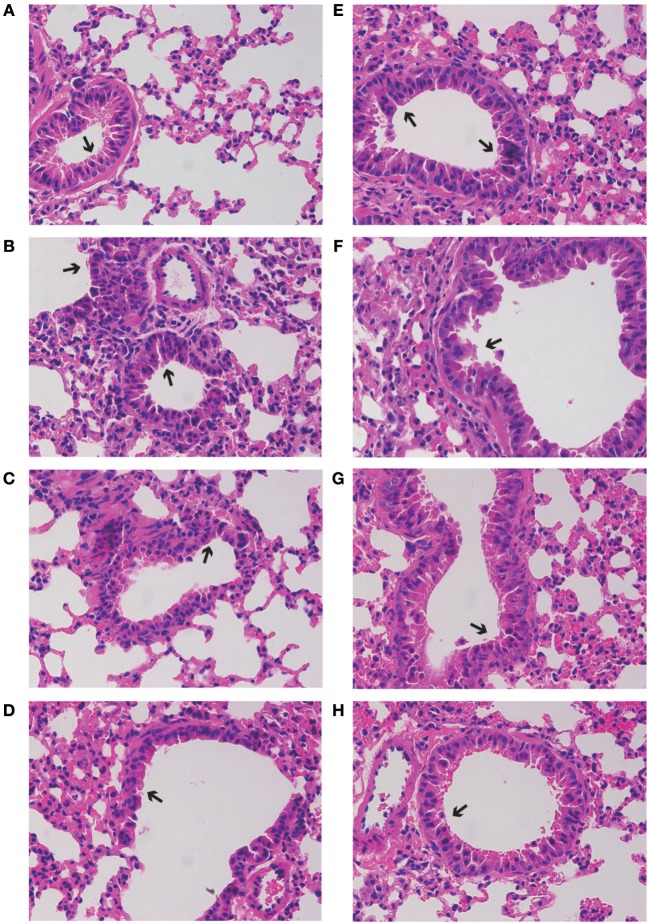
Effects of GD- mAb on RSV-induced histological changes in lung tissues using H&E method (magnification 200×) (D7, *n* = 3 in each group). **(A–H)** C group, M group, PH group, PL group, R group, TL group, TM group, and TH group.

As shown in Figure 4, severe inflammatory cell infiltration and obvious alveolar wall thickening was detected in group M (H&E staining), while GD-mAb, especially in groups PH and TH, had successfully restored lung tissues to their normal alveolar structure without significant pathological damage (Figure 4).

**Figure 5:**
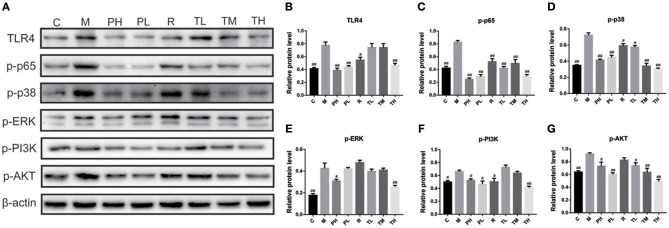
Effects of GD- mAb on phosphorylated protein levels in pathways of TLR4/NF-κB, MAPKs, and PI3K /Akt in lung using Western blot. **(A)** protein expressions of TLR4, p-p65, p-p38, p-ERK, p-PI3K, and p-AKT in C, M, PH, PL, R, TL, TM, TH groups using Western blot (D7, *n* = 3 in each group). **(B,C)** Effect of the GD- mAb on pathway of TLR4/NF-κB. **(D,E)** Effect of the GD- mAb on pathway of MAPKs. **(F,G)** Effect of the GD- mAb on pathway of PI3K/Akt. Data are presented as means ± SD. ^#^*P* < 0.05 vs. model group; ^##^*P* < 0.01 vs. model group.

As some signaling pathways, e.g., Toll-like receptors (TLRs), NF-κB, MAPKs, and PI3K/AKT, have been reported to be involved in the inflammation cascade through pathogen-associated molecular pattern recognition. Therefore, the Western blot method was used to study the expression of key proteins in these pathways. As shown in Figure 5, RSV infection significantly elevated inflammatory mediators of TLR4 (0.78), up-regulated p-p65 expression (NF-κB pathway) (0.83), p-ERK/p-p38 (MAPKs pathway) (0.43/0.72), and p-PI3K/p-AKT (PI3K/AKT pathway) (0.66/0.92) in the M group, while GD-mAb effectively inhibited further deterioration of the inflammatory response by reducing the phosphorylated protein level in the pathways of TLR4, NF-κB (p-p65), p-ERK,p-p38, and p-PI3K, p-AKTin in groups TL (0.74 vs. 0.42 vs. 0.40 vs. 0.57 vs. 0.73 vs. 0.74), TM (0.74 vs. 0.49 vs. 0.41 vs. 0.34 vs. 0.64 vs. 0.63), TH (0.47 vs. 0.30 vs. 0.25 vs. 0.31 vs. 0.41 vs. 0.49) and the prevention groups-PH (0.39 vs. 0.25 vs. 0.31 vs. 0.41 vs. 0.53 vs. 0.73) and PL (0.43 vs. 0.29 vs. 0.42 vs. 0.44 vs. 0.47 vs. 0.60) (*p* < 0.01, Figure 5).

**Figure 6:**
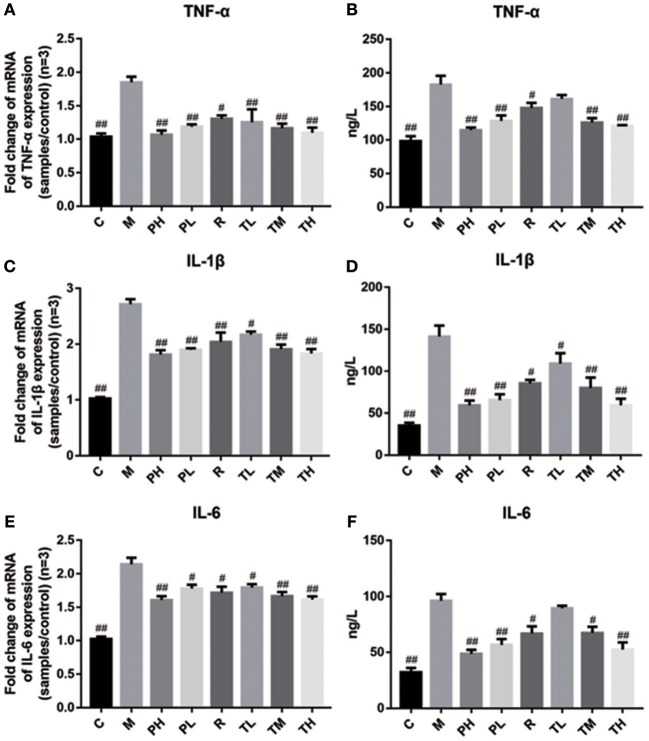
Inhibition effect of GD-mAb on inflammatory mediators of TNF-α, IL-1β, and IL-6 in mice lung tissues (D7, *n* = 3 in each group) at gene level and in serum (D7, *n* = 3 in each group) at protein level. **(A,B)** Inhibition effect of GD- mAb on TNF-α expression. **(C,D)** Inhibition effect of GD- mAb on IL-1β expression. **(E,F)** Inhibition effect of GD- mAb on IL-6 expression. Data are presented as means ± SD. ^#^*P* < 0.05 vs. model group; ^##^*P* < 0.01 vs. model group.

Finally, q-PCR and ELISA were used to investigate the release of TNF-α, IL-1β, and IL-6 in the mouse lung and serum. Consistent with the previous results, RSV greatly induced the secretion of pro-inflammatory cytokines TNF-α, IL-1β, and IL-6 in the M group (1.85 vs. 2.72 vs. 2.14), while GD-mAb significantly suppressed the release of TNF-α, IL-1β, and IL-6 in the lungs at gene level in the treatment group TL (1.25 vs. 2.17 vs. 1.80), TM (1.16 vs. 1.91 vs. 1.67), TH (1.10 vs. 1.84 vs. 1.62), the prevention group PH (1.07 vs. 1.82 vs. 1.61) and PL (1.19 vs. 1.90 vs. 1.78) compared with the M group. Similarly, GD-mAb also significantly inhibited the expression of these three pro-inflammatory factors in the treatment group TL (161.39 vs. 109.13 vs. 89.51), TM (125.79 vs. 79.75 vs. 67.20), TH (120.69 vs. 59.31 vs. 52.42), PH (114.57 vs. 59.00 vs. 48.51) and PL (128.14 vs. 65.42 vs. 56.50) in the serum at the protein level (*p* < 0.01, Figure 6).

## Discussion

RSV is still a common cause of lower respiratory tract disease and bronchiolitis in infants and young children worldwide. Up to the present day, only two FDA-approved drugs (ribavirin and palivizumab) are available for the specific prevention or treatment of RSV infection (Geevarghese and Simões, 2012). Ribavirin has limited efficacy and is seldomly used in immune compromised patients. Palivizumab is a monoclonal antibody for RSV infections in high-risk infants, and needs to be administered monthly at high cost (Yu et al., 2008). Currently, most licensed mAbs belong to chimeric or humanized mouse immunoglobulin molecules, and the allergic reactions caused by these mAbs (murine mAbs, human anti-chimeric antibody, and anti-humanized-antibody) lead to adverse outcomes and reduced their therapeutic efficacy (Stark et al., 2006; Wang et al., 2012; Guan et al., 2013). Thus, the development of fully human mAbs with more potent neutralizing activities remains a key goal for antibody research.

In the present study, phage display and antibody engineering were used to develop the GD-scFv and GD-mAb against RSV GD protein, and both these proteins showed a high affinity and high specificity to antigen GD (Figures 1, 2), while GD-mAb also exhibited high neutralizing activity through plaque reduction virus neutralization assays *in vitro* (Figure 2). We report that high specificity and high affinity ensures the affinity of the antibody to find and combine to the target protein, and the high neutralizing activity ensures its high killing effects on the virus.

To further evaluate the therapeutic effect of GD-mAb, we employed a mouse model of RSV infection. Our results indicate that GD-mAb significantly decreases the pulmonary inflammatory response and decreases viral titer in the lungs by blocking cell attachment and intracellular replication (Shi et al., 2017), suppressing inflammatory cell infiltration and alleviating lung injury (Figures 3–6). Toll-like receptors (TLRs) are innate immune receptors expressed by immune cells, and they are positioned as a first line of innate defense by recognizing pathogen-associated molecular patterns as well as endogenous signals of tissue injury (Nahid et al., 2011; Steensma et al., 2014). In previous studies, researchers found that the increase of TLR4 on the cell surface plays a fundamental role in regulating the immune response against RSV infection. Moreover it could activate NF-κB members (Kurt-Jones et al., 2000; Monick et al., 2003; Gagro et al., 2004), involving the expression and release of several cytokines and chemokines that were regulated by NF-κB. Furthermore, a recent study has shown that RSV activation of p38 in mitogen-activated protein kinase pathways (MAPKs) in a TLR4 mediated manner during the early stage of infection, utilized p38 to enter the cell for replication (Marchant et al., 2010). Researches demonstrated that PI3K/AKT could regulate the activation of multiple intracellular signaling cascades and is a function of many inflammatory mediators (Xuan et al., 2017). TLR4, MAPKs and P13K/ATK could also lead to an increase in the nuclear translocation and transcriptional activity of NF-κB (Hattori et al., 2003; Capiralla et al., 2012). When the NF-κB pathway was activated, massive amounts of pro-inflammatory cytokines (e.g., TNF-α, IL-1β, and IL-6) were released, which were strongly associated with the outcome of inflammatory disease (Ren et al., 1996). In our work, RSV infection greatly enhances the yield of TLR4, phosphorylated protein p38, ERK, PI3K, and AKT in the MAPKs and PI3K/Akt pathways, which then promotes the phosphorylated protein p65 (NF-κB pathway), while GD-mAb effectively inhibits the further deterioration of the inflammatory response by reducing their phosphorylation in lung (Figures 5, 6).

In addition, accumulating evidence indicates that viral invasion involves the production of a plethora of cytokines, chemokines, and immune modulatory mediators, including TNF-α, IL-1β, and IL-6 which can strongly influence inflammatory responses. As we know, TNF-α is an important inflammatory cytokine in the development of lung inflammation and higher levels of TNF-α are associated with chronic obstructive pulmonary disease (Lewis, 2007; Luo et al., 2017). IL-1β is a crucial inflammatory factor involved in pneumonia and research indicates that the level of IL-1β in the lungs of chronic obstructive pulmonary disease patients is higher than that of non-smokers (Pauwels and Rabe, 2004). In addition, studies indicate that a benefit is obtained when the IL-6 response is suppressed in mice challenged with RSV. Therefore, the low expression of TNF-α, IL-1β, and IL-6 in mice in the pre-treated and treated groups indicate that GD-mAb is beneficial in reducing inflammation in RSV-infected mice.

Our data indicate that the anti-RSV GD-mAb possesses a high affinity and high specificity with the GD protein, and can effectively neutralize RSV *in vitro*. The animal results further indicate that GD-mAb can significantly lower the lung index and reduce the lung virus titer in mouse lungs and effectively reduce the phosphorylated protein level in pathways of TLR4/NF-κB, MAPKs, and PI3K/Akt, which inhibit the release of pro-inflammatory factors TNF-α, IL-1β, and IL-6 in mouse serum and lungs. These findings suggest that GD-mAb may be a potential agent for the prevention and treatment of RSV infection. However, the qualitative results of H&E staining, the semiquantitative assays of Western blotting, and the absence of a positive control (e.g., Palivizumab) reduce the reliability of our work, and further work is needed to explore the actual effect of GD-mAb on RSV infection.

## Ethics Statement

The present study was approved by the Ethical Committee of the Second Affiliated Hospital of Nanchang University (Nanchang, China) and all methods were performed in accordance with the approved guidelines.

## Author Contributions

TC designed the experiment. PT, YW, HL, YY, XW, and HW performed the experiments. TC and PT analyzed the data and wrote the manuscript. All authors discussed the results and commented on the manuscript.

### Conflict of Interest Statement

The authors declare that the research was conducted in the absence of any commercial or financial relationships that could be construed as a potential conflict of interest.
